# Suspension cultivation of mosquito cell lines for the production of the mosquito-borne flavivirus Usutu virus in a stirred-tank bioreactor

**DOI:** 10.1038/s41598-025-33792-z

**Published:** 2026-01-09

**Authors:** Dennis Kenbeek, Joshua Kanters, Karim Bousnina, Mels Schrama, Dirk E. Martens, Jelke J. Fros

**Affiliations:** 1 Laboratory of Virology, Wageningen University and Research, Wageningen, The Netherlands; 2 Bioprocess Engineering, Wageningen University and Research, Wageningen, The Netherlands

**Keywords:** Biological techniques, Biotechnology, Microbiology

## Abstract

Mosquito-borne pathogens are a global health burden. The realization of a scalable serum-free mosquito cell suspension cultivation method would enable the production of novel biotechnological products, including vaccines, recombinant proteins, and (modified) viruses for biological control. This study reports the adaptation of two *Aedes*-derived cell lines, C6/36 and U4.4, to serum-free suspension conditions using the chemically defined EX-CELL medium. Both cell lines achieved high cell concentrations (> 1.0 × 10^7^ cells/mL) in shake flasks with minimal adaptation. The C6/36 suspension cultures were subsequently transferred to a 500 mL stirred tank bioreactor (STR), which likewise achieved high cell concentrations (1.3 × 10^7^ cells/mL) and a doubling time of 31–36 h. As a proof of concept for the production of a virus in this system, C6/36 cells were cultivated in a STR and infected with the mosquito-borne flavivirus Usutu virus (USUV). These cultures produced high virus titers (> 3.6 × 10^8^ TCID_50_/mL), with similar growth kinetics compared to adherent and shake flask cultures. This work is the first to show C6/36 mosquito cell cultivation in a bioreactor and demonstrates the potential of this serum-free suspension culture system for the efficient production of mosquito-infecting viruses.

**Supplementary Information:**

The online version contains supplementary material available at 10.1038/s41598-025-33792-z.

## Introduction

Arthropod-borne viruses (arboviruses) are viruses transmitted by blood-feeding arthropods such as mosquitoes and ticks. These viruses pose a significant global health risk, infecting hundreds of millions of people worldwide each year^1^. Among the most prevalent are mosquito-borne viruses such as the flaviviruses dengue virus (DENV), Zika virus (ZIKV), West Nile virus (WNV), and yellow fever virus (YFV), as well as alphaviruses like chikungunya virus (CHIKV) and Venezuelan equine encephalitis virus (VEEV). These viruses are transmitted to vertebrate hosts by mosquito vectors, primarily from the *Aedes* and *Culex* genera. Due to changes in the ecological landscape, including urbanization and climate change, these mosquitoes and their viruses continue to spread worldwide. Despite the growing incidence of arbovirus infections, only a few licensed vaccines are currently available, most of which rely on traditional live-attenuated or inactivated virus platforms^2^. These vaccines are produced either in embryonated eggs (e.g., the classical YFV 17D live-attenuated vaccine) or in mammalian cell culture platforms (e.g., Vero, HEK293, CHO cells)^3–14^. Although these production platforms have been successful, a new generation of arbovirus vaccines is not always compatible with existing production methods.

Recent advancements in arbovirus vaccine development have capitalized on the unique dual-host life-cycle of arboviruses. For instance, multiple groups have developed vaccines against several arboviruses (CHIKV, ZIKV, JEV, and WNV) through generating chimeric viruses with naturally occurring insect-specific flaviviruses or alphaviruses^15–18^. These chimeric viruses display arbovirus antigens, whilst containing insect-specific viral replicases, which makes them unable to replicate in vertebrate cells. This results in safe vaccines that can be grown to high titers in mosquito cells^15,16^. Furthermore, these chimeric viruses enable rapid and safe production of antibody based diagnostics^16,19^. Another promising approach involves the introduction of mutations into mosquito-borne viruses to achieve specific attenuation in vertebrate cells^20–25^. Therefore, these new methods exploit the dual-host phenotype of arboviruses by maintaining high levels of replication in mosquito cells, while replication in mammalian cells is either strongly attenuated^20–25^ or completely absent^15–18^. Mosquito cell cultures thus present an attractive method for the production of these next-generation arbovirus vaccines. However, no effective production method utilizing mosquito cells is currently available.

One of the most widely used mosquito cell lines is C6/36, derived from *Aedes albopictus* larvae^26^. C6/36 cells lack a functional RNA interference (RNAi) machinery, rendering them highly susceptible to a wide range of arboviruses and capable of producing high virus titers^27,28^. These properties make C6/36 cells excellent candidates for an arbovirus vaccine production platform. However, the lack of a functional RNAi system can severely reduce cell viability upon infection^29^. Another commonly used mosquito cell line, U4.4, originates from the same parental cell line as C6/36, and does contain a functional RNAi pathway, which may extend the potential production time compared to C6/36^30–32^. All currently available mosquito cell lines rely on adherent cultivation methods. Although larger scale adherent cultivation is possible using roller bottles or cell factories, scale-up is less effective due to surface area limitations, and labor-intensive attachment and detachment procedures. Microcarriers are often used to allow for cultivation in a stirred-tank bioreactor (STR), but these still suffer from detachment and reattachment difficulties as well as sensitivity to shearing and have limited agitation rates and mixing at larger scales^4^. Instead, direct suspension cultivation in a STR offers favorable scalability by allowing increased culture volumes, simplified passaging procedures, higher culture densities, and enabling better control of culture parameters through agitation. Furthermore, existing mosquito cell lines typically require fetal bovine serum (FBS) for growth, introducing regulatory and contamination risks associated with animal-derived components. Additionally, serum can have high batch-to-batch variability, high costs, and may raise ethical concerns^33–35^. To address these issues, suspension-adapted mosquito cell lines capable of growing in serum-free conditions are essential. Only a limited number of studies describe the suspension cultivation of mosquito cell lines^36–39^. Suchman and Carlson (2004) were the first to adapt C6/36 cells to suspension culture in spinner flasks, for which they used serum-free Sf900-II medium and demonstrated successful production of densovirus in this system^39^. Recently, Dawurung et al. described an innovative strategy where C6/36 cells were adapted to Sf900-III serum-free medium, allowing them to be cultivated in shake flasks to high cell concentrations (> 2.5 × 10^7^ cells/mL)^36^. The cultivation phase was then followed by a medium exchange to the chemically defined FortiCHO medium for the production of flavivirus vaccine candidates^36^. This medium exchange is necessary as Sf900-III medium proved too acidic for the production of flaviviruses^36^. Although successful, this strategy required adaptation of C6/36 cells to the medium exchange conditions, as this medium is not optimized for insect cells. Furthermore, CD-FortiCHO requires CO_2_ for pH buffering, complicating the cultivation method. This method has not yet been tested in bioreactor systems, as the studies remained restricted to shake flasks.

These studies have so far focused on C6/36 cells, whereas cultivation of an RNAi competent cell line may prove beneficial and can expand possible applications. In this study, we aimed to investigate the potential of C6/36 and the related U4.4 cell line to grow in chemically defined serum-free suspension conditions, and to study how mosquito infecting (arbo)viruses replicate under these conditions.

We report protocols for cultivating these cell lines in serum-free chemically defined suspension culture conditions using EX-CELL medium. We show that both cell lines can be grown effectively in shake flasks, and with C6/36 cells we developed the protocols to cultivate these cells in a STR. Additionally, we assessed the effects of these new serum-free chemically defined culture conditions on flavivirus production, using Usutu virus (USUV) as a model mosquito-borne flavivirus^40^. USUV is a close relative to pathogenic viruses such as WNV and JEV and is transmitted between birds by vector mosquitoes. While infections in humans and other mammals do occur, severe USUV disease in humans is uncommon^40^. To our knowledge, this is the first study where serum-free suspension cultivation of mosquito cells was shown in a chemically defined medium (EX-CELL), as well as the cultivation of C6/36 cells in a STR for the production of a flavivirus.

## Results

### The effect of serum-free EX-CELL medium on virus production

**Fig. 1:**
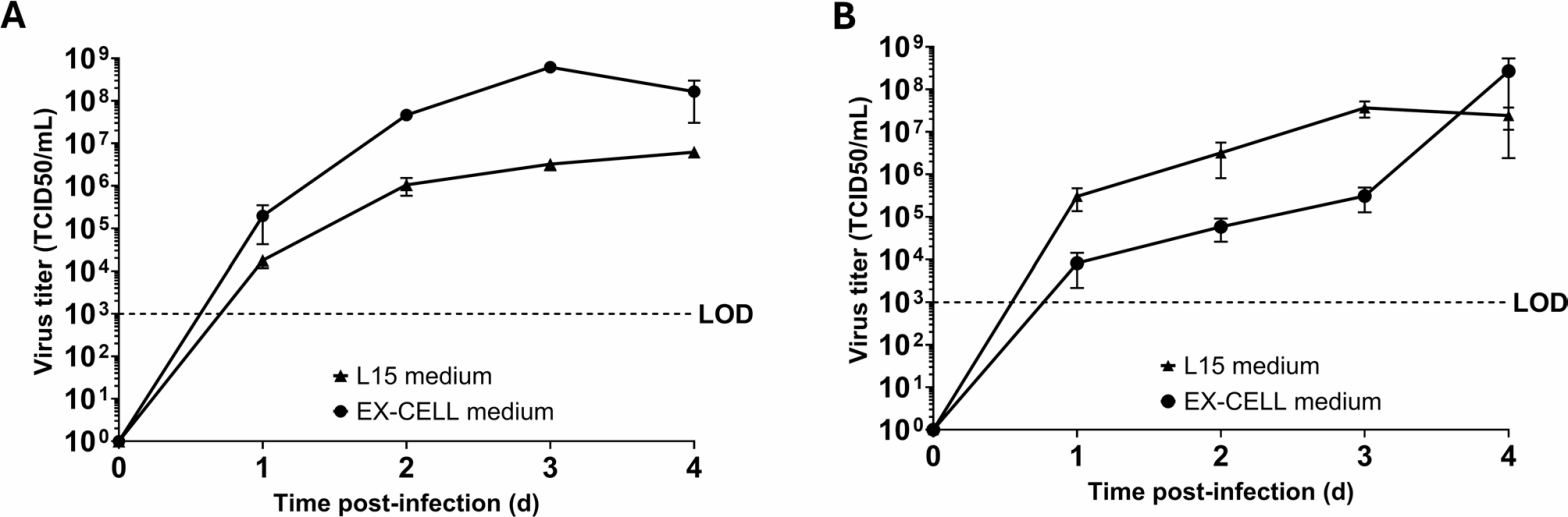
Virus replication in adherent mosquito cells cultivated in either L-15 or chemically defined medium. (**A**) Adherent C6/36 cultures were infected at an MOI of 0.1 TCID_50_/cell with USUV in either L-15 or EX-CELL medium. (**B**) Adherent U4.4 cells were infected with USUV at an MOI of 0.1 TCID_50_/cell in either L-15 or EX-CELL medium. Each datapoint represents the average of three independent experiments (*n* = 3), and error bars represent the standard error of the mean.

Cell culture medium can influence virus replication and stability, especially for flaviviruses, where pH affects the infectivity of the virion^36,41,42^. To study the impact of serum-free chemically defined EX-CELL medium on flavivirus production in C6/36 and U4.4 mosquito cells, and whether it would be compatible with the production of (arbo)viruses, we first compared infections in adherent cell cultures in EX-CELL medium to those in L-15 medium. To ensure equal numbers of infected cells, C6/36 and U4.4 cells were infected with USUV (MOI 0.1 TCID_50_/cell) in L-15 medium for 3 h at 27 °C, after which the medium was replaced with either EX-CELL medium or fresh L-15 medium. Infected C6/36 cells that were incubated in EX-CELL medium demonstrated a remarkable > 100-fold increase in virus production compared to cells in L-15 medium (6.20 × 10^6^ TCID_50_/mL in L-15 and 6.20 × 10^8^ TCID_50_/mL in EX-CELL) (Fig. 1A). In contrast, U4.4 cells infected in EX-CELL medium exhibited slower virus progression in the first days post-infection compared to those cultured in L-15 medium (Fig. 1B). Nevertheless, both conditions resulted in similar peak virus titers (3.67 × 10^7^ TCID_50_/mL in L-15 and 2.67 × 10^8^ TCID_50_/mL in EX-CELL). These results indicate that EX-CELL medium can facilitate the production of high titers of infectious virus and can even outperform traditional L-15 medium during infections in C6/36 mosquito cells.

### Adaptation of mosquito cells to serum-free suspension cultivation

**Fig. 2:**
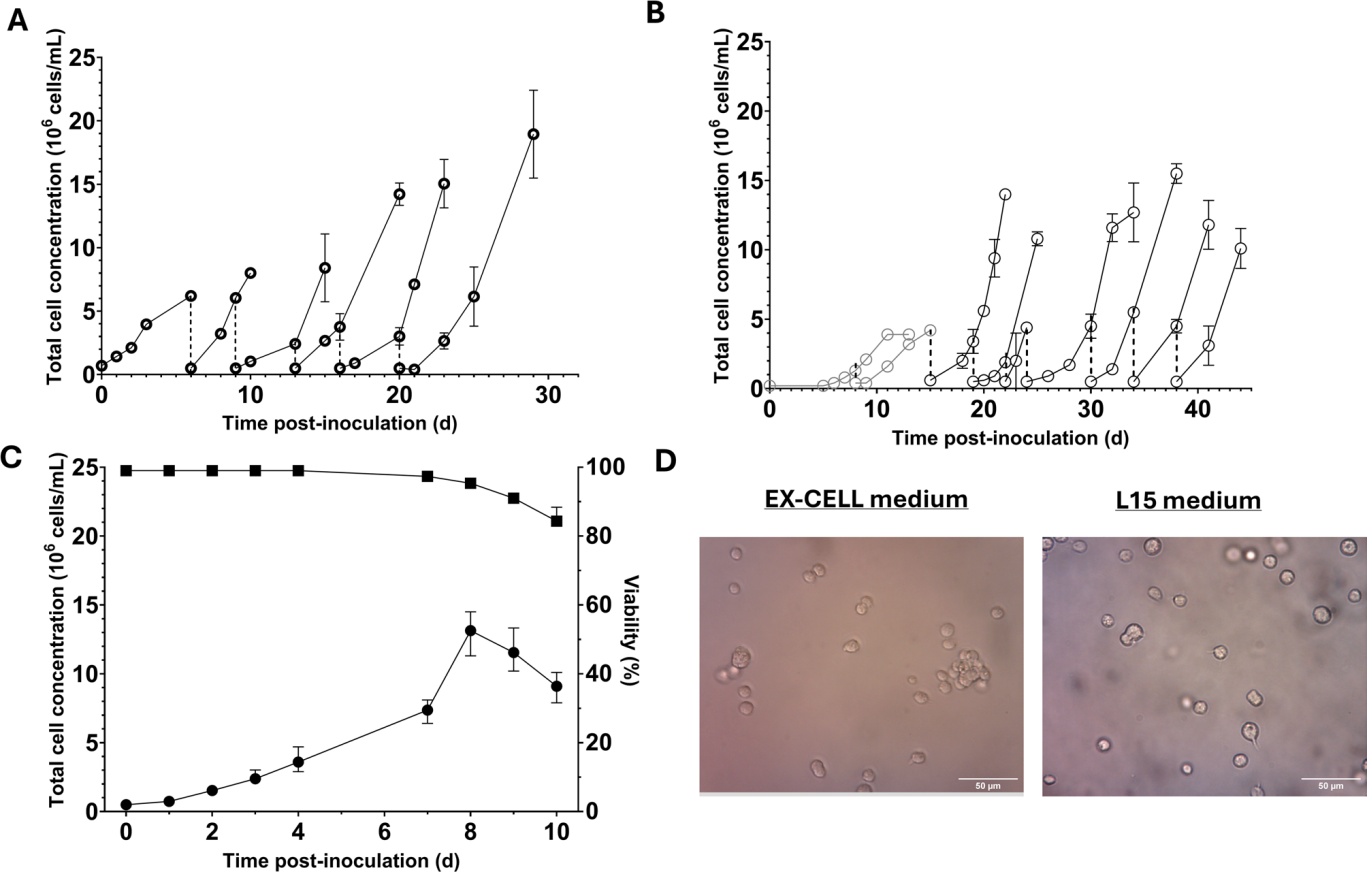
The adaptation of C6/36 cells to serum-free suspension conditions using EX-CELL medium. (A) Adaptation of C6/36 cells from an adherent culture by seeding cells at 0.7 × 10^6^ cells/mL in 10 mL. After two passages (grey) the culture was split into two duplicate cultures (black). (B) Revival of a frozen cell bank of suspension-adapted C6/36 cells. The first two passages are from a single culture and represent recovery (grey). The cell concentration of subsequent passages represent the average of two replicate cultures (black). The average and standard deviation of the duplicate cultures are shown (*n* = 2). The dotted line shows a passaging event. (C) Characterization of C6/36 growth in serum-free suspension (*n* = 3). (D) Example image of C6/36 cell morphology in EX-CELL and L-15 medium after detachment from an adherent culture. Error bars indicate the standard error of the mean.

To adapt C6/36 cells to suspension cultivation in EX-CELL medium, cells were transferred from an adherent culture (90% confluent T25 flask), cultivated in standard L-15 medium to EX-CELL medium in suspension (0.70 × 10^6^ cells/mL in 10 mL), and cultured at 27 °C at 110 RPM shaking in a 125 mL shake flask. The first suspension culture consisted of a ratio of 40:60 L-15 medium to EX-CELL medium, which was reduced through passaging, where new passages were started whenever a total cell concentration of > 2.0 × 10^6^ cells/mL was measured. This resulted in < 1% L-15 medium after only 3 passages. The original culture was also monitored for several days post-passaging to allow for further monitoring of the growth. New passages were seeded at a concentration of 0.50 × 10^6^ cells/mL in a total volume of 20 mL. After two passages, the culture was split into two duplicate cultures. The first two passages showed limited growth but recovered quickly in subsequent passages (Fig. 2A), and reached a maximum cell concentration of 1.90 × 10^7^ cells/mL at passage 6 (Table 1). To further evaluate the growth characteristics of C6/36 cells under suspension conditions and whether they are effectively revived from a cryopreserved cell bank, C6/36 cells were frozen after 18 passages. Following revival, the cryopreserved cells demonstrated rapid recovery, becoming stable after just two passages (Fig. 2B). The culture was then split into two parallel suspension cultures, which were passaged seven times and showed stable growth throughout. Both replicate lineages displayed comparable growth rates with average specific growth rates of 0.50 ± 0.08 (d^−1^) (33 h doubling time) for the first and 0.44 ± 0.08 (d^−1^) for the second lineage (38 h doubling time). These values are similar to doubling times observed for adherent cultures in our lab (~ 35 h) and within the described culture parameters^43^. Furthermore, all passages reached consistently high cell concentrations across passages (> 10^7^ cells/mL). The highest total cell concentration observed was 1.55 × 10^7^ cells/mL. To characterize the growth within a single passage, the cryopreserved cells were revived, passaged three times, and then divided into three parallel cultures that were monitored for 10 days (Fig. 2C). These cultures reached a plateau at 8 days with a maximum average total cell concentration of 1.31 × 10^7^ and a growth rate of 0.41 ± 0.03 (d^−1^) (Table 1), after which a gradual decrease in cell number and viability was observed. Furthermore, the morphology of C6/36 cells in EX-CELL suspension was similar to that of freshly detached cells from standard adherent cultures in L-15 medium. However, small clumps of cells were occasionally observed in the suspension cultures (Fig. 2D), with these clumps increasing in size as higher cell concentrations were attained. These findings suggest that C6/36 cells readily adapted to cultivation in serum-free EX-CELL medium.

**Fig. 3:**
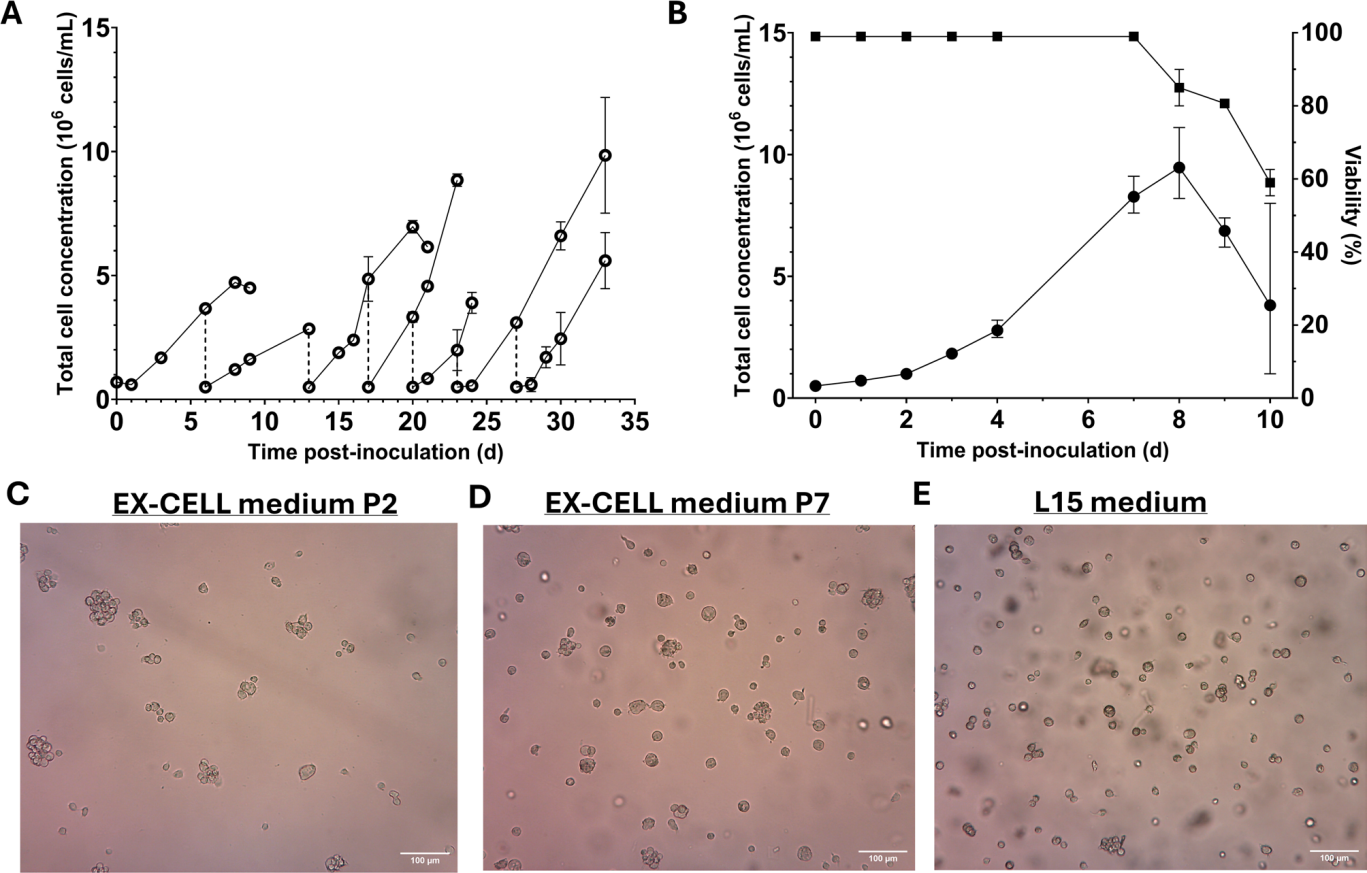
The adaptation of U4.4 cells to suspension cultivation in EX-CELL medium (**A**) U4.4 cells were adapted to suspension culture in EX-CELL medium from an adherent culture grown in L-15 medium by inoculating at 0.7 × 10^6^ cells/mL in 10 mL. The first two passages are a single culture (grey), which was split into two duplicate cultures (black). The dotted line shows a passaging event. (**B**) Characterization of U4.4 growth in serum-free suspension conditions (*n* = 3). (**C**) Example of U4.4 cell morphology in EX-CELL in passage 2 and (**D**) passage 7 compared to (**E**) the morphology of freshly detached U4.4 cells cultivated in an adherent flask with L-15 medium. Error bars indicate the standard error of the mean.

Next, we investigated whether the RNAi competent U4.4 cells could similarly adapt to serum-free suspension conditions. An adherent U4.4 culture grown in L-15 medium was transferred to serum-free EX-CELL medium under suspension conditions with 0.70 × 10^6^ cells/mL in 10 mL, resulting in a 40:60 ratio of L-15:EX-CELL medium. The amount of L-15 medium was reduced by passaging, resulting in less than 1% L-15 medium at passage 4. After two passages, the culture was split into two duplicate cultures. The suspension cultures were passaged when total cell concentrations of 3–6 × 10^6^ cells/mL were reached. Similar to the adaptation of the C6/36 cells, the first two passages grew slowly but then recovered in subsequent passages (Fig. 3A). The maximum total cell concentration achieved during passaging was 1.15 × 10^7^ cells/mL. To further characterize the growth of a single suspension culture, a growth curve was made after three passages by splitting a single culture into three parallel cultures (Fig. 3B). This showed that the U4.4 cells reached a plateau at 8 days and reached an average total cell concentration of 9.47 × 10^6^ cells/mL and a growth rate 0.37 ± 0.04 (d^−1^) (45 h doubling time) (Table 1). Furthermore, the U4.4 cells showed clumping behavior similar to C6/36 cells (Fig. 3C), and after several passages, enlarged cells were observed (Fig. 3D). Despite these enlarged cells and the observed clumping behavior, the viability remained high throughout the passaging, and cell morphology was overall similar to that of U4.4 cells detached from a cell culture flask (Fig. 3E).

**Table 1:** Measured cell concentrations and growth rate of C6/36 and U4.4 cells during adaptation and characterization.

Cell line	Maximum total cell concentration during adaptation (x10^7^ cells/mL)	Maximum total cellconcentration growth curve (x10^7^ cells/mL)	Specific growth rate µmax (d^−1^)	Doubling time (h)
C6/36	1.90	1.31	0.41 ± 0.03	40
U4.4	1.15	0.95	0.37 ± 0.04	45

The specific growth rate was calculated from the growth curve.

### Serum-free suspension cultivation in a stirred-tank bioreactor

**Fig. 4:**
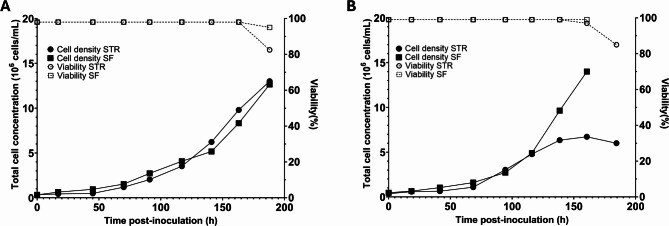
Cultivation of C6/36 cells in a stirred-tank bioreactor and a shake flask originating from the same culture. Cell concentration (left axis) and viability (right axis) of C6/36 cells grown in an STR and a shake flask (SF) control, for both the first (**A**) and second (**B**) bioreactor experiment.

After successfully adapting C6/36 cells to suspension cultivation in shake flasks, we next assessed whether the cells could be transferred to a STR (500 mL). Two independent STR runs were performed, where each consisted of a STR and shake flask control culture originating from the same inoculant. Each reactor run showed similar growth kinetics to their shake flask control, especially in the early phase (Fig. 4A, B). The first STR run exhibited growth kinetics nearly identical to the shake-flask culture, with both achieving a specific growth rate of 0.019 h^−1^ (doubling time ~ 36 h) (Table 2). Neither culture reached a plateau during the 8-day experiment (Fig. 4A). The second bioreactor run initially showed a comparable growth rate (0.022 h^−^^1^) to the first run (0.019 h^−1^) and began to plateau after 117 h, resulting in a final cell concentration of 6.0 × 10^6^ cells/mL compared with 1.3 × 10^7^ cells/mL in the first run (Table 2; Fig. 4B). Despite the differences in final cell concentrations between the two STR runs, the viabilities at the end of both cultures were only slightly lower than those of the shake flask controls and remained above 80% (Fig. 4A, B). The medium pH remained relatively stable during the first 100 h of both runs before gradually increasing thereafter (Supplementary Fig. 1).

Cell clumps were observed both in the STR and shake flask C6/36 cultures, similar to those in Fig. 2D. Because cell aggregates can be more susceptible to shear stress, we estimated the Kolmogorov length in the STR to be approximately 123 μm. Visual inspection suggests most clumps had a diameter smaller than 123 μm, indicating that most aggregates would not have encountered appreciable shear forces. This was further confirmed by the absence of substantial cell death, as demonstrated by the high viability observed in the first STR experiment. Although further optimization is needed to improve consistency between runs, these findings highlight the feasibility and potential of cultivating C6/36 cells in stirred-tank bioreactors.

**Table 2:** Summary of cell growth in STRs and shake flasks of C6/36 cells.

Culture	Final total cell concentration (x10^7^ cells/mL)	Specific growth rate (h^− 1^)	Doubling time (h)
STR 1	1.30	0.019	36
Shake flask 1	1.26	0.019	36
STR 2	0.60	0.022	31
Shake flask 2	1.40	0.021	33

### Flavivirus production in a STR

**Fig. 5:**
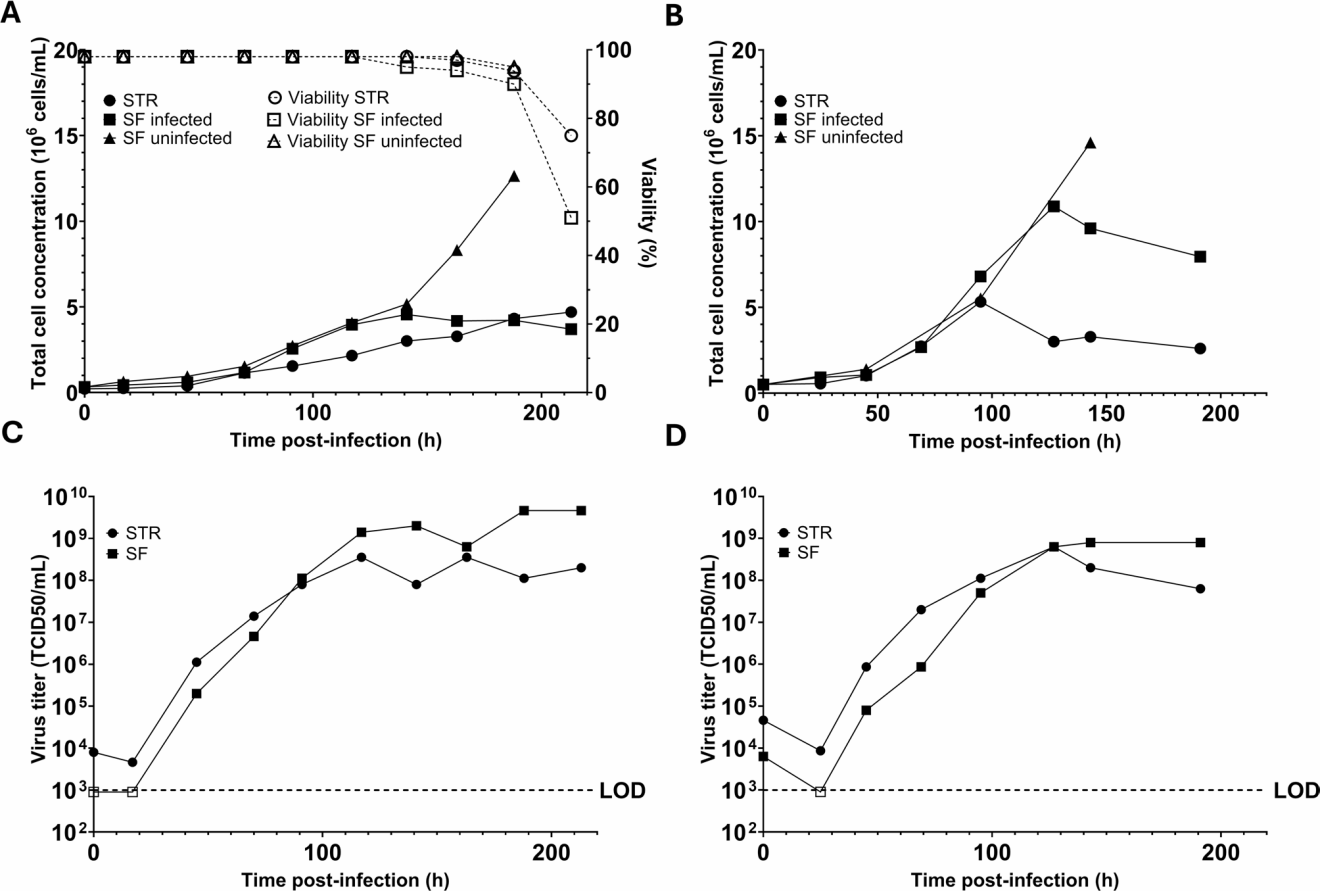
Virus production using C6/36 cells inside a STR and shake flasks. (**A**, **B**) C6/36 cell concentration of two independent STR runs with virus infection. As controls, a shake flask without or with infected C6/36 cells was included. In the first reactor run, viability data is shown in dashed lines. (**C**, **D**) Viral titers produced in the STR and SF from both independent experiments. LOD = Limit of detection. Error bars indicate the standard error of the mean. Virus titers below the detection limit are depicted on the LOD line and indicated with an open instead of a solid data point.

Given the high virus production of C6/36 cells in EX-CELL medium and their successful cultivation in an STR, we next evaluated this system for flavivirus antigen production using a replicating flavivirus as a proof of concept. Two independent STR C6/36 cultures and a corresponding shake flask control originating from the same inoculum, were infected with USUV at an MOI of 0.1 TCID_50_/cell. An additional shake flask with uninfected cells was included to assess the impact of the USUV infection on C6/36 cell growth. Initially, the infected C6/36 cultures exhibited growth comparable to their uninfected counterparts in both the STR and the shake flask (Fig. 5A, B). In the first bioreactor experiment, the cell growth started to slow down after 70 h post-infection compared to the uninfected shake flask. In the infected shake flask, a decline in growth occurred after 144 h (Fig. 5A). Although a decline in growth was observed, cell viability only started to decline after 160 h post-infection (Fig. 5A). Both the infected shake flask and the bioreactor reached a similar final total cell concentration (Table 2), which was considerably lower than that of the uninfected shake flask, indicating a strong impact of the infection on the cells. The second experiment showed a similar trend, with both the infected and uninfected cultures growing at comparable rates during the initial stage, followed by the infected cultures plateauing earlier and reaching a lower final cell concentration than the shake flask with uninfected cells (Table 2) (Fig. 5B).

Although USUV infection caused a reduction in cell concentrations compared to uninfected controls, both the STR and shake flasks experiments successfully produced high viral titers, with a peak titer of 3.56 × 10^8^ TCID_50_/mL achieved after approximately 160 h post-infection in the first STR and 6.32 × 10^8^ TCID_50_/mL after 130 h in the second STR (Fig. 5C, D). These titers are comparable to the peak titers obtained in the adherent cultures (average of 6.20 × 10^8^ TCID_50_/mL) (Fig. 1A). The yields per cell at peak virus titers were 1.09 × 10^2^ TCID_50_/cell and 2.11 × 10^2^ TCID_50_/cell for the first and second STR runs respectively. In comparison, the first shake flask culture reached a higher yield of 1.09 × 10³ TCID₅₀/cell, whereas the second shake flask produced a similar yield to the STR runs at 8.29 × 10^1^ TCID_50_/cell (Table 3). The overall infections in suspension were slower to reach peak titers compared to adherent cultures. These findings indicate that virus infection in suspension mosquito cultures and STRs is a feasible strategy to scale-up production of viral antigens.

**Table 3:** Summary of cell growth and virus production in the STRs and corresponding shake flasks.

Culture	Final total cell concentration (x10^7^ cells/mL)	Total cell concentration at peak virus titer (x10^7^ cells/mL)	Peak virus titer (TCID_50_/mL)	Yield (TCID_50_/cell)
STR 1	0.40	0.33	3.56 × 10^8^	1.09 × 10^2^
Shake flask 1	0.20	0.42	4.62 × 10^9^	1.09 × 10^3^
Uninfected shake flask 1	1.30	–	–	–
STR 2	0.26	0.30	6.32 × 10^8^	2.11 × 10^2^
Shake flask 2	0.80	0.96	7.96 × 10^8^	8.29 × 10^1^
Uninfected shake flask 2	1.50	–	–	–

Yield is defined as the virus produced per cell at peak virus titer (TCID_50_/cell).

## Discussion

The realization of a serum-free chemically defined suspension culture system for mosquito cells would allow for the production of a wide range of products, such as viral antigens and recombinant proteins produced by mosquito(-borne)viruses, insect-specific viruses for vector control, as well as a new generation of arbovirus vaccines. Efforts to cultivate C6/36 in suspension have been successful, but cultivation of other mosquito cell lines, as well as transfer from shake flasks to bioreactors has not yet been realized^36–39^. In this study, we assessed the adaptation of two commonly used mosquito cell lines, C6/36 and U4.4, to growth in suspension using serum-free chemically defined conditions in shake flasks. Furthermore, the capability of C6/36 to be cultivated in a stirred tank bioreactor, as well as the production of USUV under these conditions, was assessed.

Both mosquito cell lines showed a relatively straightforward adaptation from an adherent culture to a suspension culture by transfer from an adherent culture, requiring minimal adaptation. Each cell line also showed high cell concentrations (C6/36 ~ 1.9 × 10^7^ cells/mL and U4.4 1.15 × 10^7^ cells/mL) and doubling times comparable to adherent C6/36 and U4.4 cultures (~ 35 h). These doubling times are comparable to what has previously been reported for C6/36 cells^36^ in shake flasks, and the culture concentrations are similar to the commonly used *Spodoptera frugiperda* insect cell lines^44^. However, the U4.4 cell line displayed a slower growth rate and reached lower maximal cell concentrations than C6/36 (Table 1). USUV infections were also slower in U4.4 cells and resulted in lower titers. Nevertheless, the U4.4 cell line showed promising results and adapted rapidly to EX-CELL medium. Higher viral titers are often seen in C6/36 cells because they are extremely permissive to arbovirus infections due to their nonfunctional RNAi pathway^27^. However, some viral species, including insect-specific viruses used in chimeric vaccine development against human and animal pathogens (e.g., WNV, JEV), strongly reduce the viability of mosquito cell cultures in the absence of a functional RNAi pathway^29,45^. Therefore, we hypothesize that U4.4 cells with a functional RNAi pathway may prove particularly useful to replicate these otherwise cytopathic viruses. Specifically comparing insect-specific virus replication in U4.4 and C6/36 cells is a topic for future investigations.

Both cell lines showed clumping behavior during suspension cultivation. Clumping in suspension cultures can be suboptimal as it may result in nutrient and oxygen limitation for the cells in the center, which can result in cell death^46,47^. In the context of a virus infection, this may result in less efficient virus infection^48^. Although for some viruses cell-to-cell contact is reported to enhance viral spread^49^. Therefore, it will be interesting to study whether methods to minimize cell clumping, e.g., through the use of an anti-clumping agent or increasing agitation rates^36,46,50^, may improve flavivirus infection in suspension mosquito cells in the future. Moreover, when applying anti-clumping measures, the effect on virus particles has to be considered. In this study pluronic was used, but this may have affected USUV binding to cellular receptors as well. Nevertheless, both cell lines reached high cell concentrations (> 10^7^ cells/mL) in shake flasks and C6/36 additionally in the STR (300 mL). Furthermore, the independent repeats showed that the process is reproducible in shake flasks, whereas STR experiments deviated after 117 h post-inoculation in one of the reactors. Although this provides a proof-of-concept, this system still requires optimization to improve reproducibility. To this end, it will be imperative to study how different parameters (e.g., metabolites, media composition, or pH) affect cell replication and viability. Moreover, it would also be interesting to introduce suspension C6/36 cells to other types of bioreactors and compare different production systems.

To determine the ability of a serum-free suspension mosquito-cell platform for the production of viruses, we performed infections with USUV as a model. Medium composition can affect virus production, especially for flaviviruses that are sensitive to pH^51^, as well as the presence of serum can impact virus production^52,53^. Therefore, we first quantified the effect of the EX-CELL medium on virus production in adherent cells. Interestingly, C6/36 cells increased (> 100x) USUV production using EX-CELL medium compared to L-15 medium. It is possible that the diversity of biological components in serum affected the infectivity of viral particles in the medium. Other studies have shown that removal of serum can increase virus replication, as serum may prevent virus adsorption onto host cell receptors^52,53^. However, infections of U4.4 cells did not show increased virus replication in EX-CELL medium compared to L-15 medium. Investigating the underlying reason for the observed change in virus production with the different media could be interesting to further fine-tune media composition for improved virus production. The increased virus production in EX-CELL medium, combined with the ability to produce C6/36 in a STR, makes C6/36 an excellent candidate for large-scale virus production. Indeed, STR reactor experiments with USUV infections of C6/36 cells demonstrated virus production across experiments with very similar virus titers (> 10^8^ TCID_50_/mL, Table 3). During virus production in STRs, the cell concentrations were lower than those in the shake flask control containing uninfected C6/36 cells. This is likely driven by virus-induced cytopathic effects and shows that optimizing the timing of infection can be important to improve virus production. Furthermore, other parameters such as the MOI, DO, pH, medium composition, timing of harvest, or multiple virus harvests can still be investigated to further optimize the process and increase yields of the virus produced. For flaviviruses especially, the pH is important^36^, but even DO levels have been reported to influence viral replication, as viruses have evolved to infect at tissue-specific oxygen levels^1^. For example, tissues within the body usually have low DO levels (1–13%, most oxygen is carried in red blood cells), and DENV infectivity is reported to be higher within this hypoxic environment^55,56^. DO-levels could thus be an important parameter for the optimization of virus production, which is likely dependent on the virus.

This proof-of-concept production of USUV thus demonstrates the utility of this system, which can be applied to a wide range of applications. In particular, the production of chimeric vaccines utilizing insect-specific viruses as backbone^16–18,36,54^, large-genome recoding-based attenuated viruses^21,25,55^, as well as insect-specific viruses for vector-and insect control^29,39,45^, will be facilitated as these cannot be (efficiently) produced in vertebrate cell lines. However, mosquito cell lines often harbor persistent insect-specific viruses, which may not only impact regulatory approval, but can also have an impact on virus production. Yet, a study by Weger-Lucarelli et al. did not find any RNA viruses in C6/36 cells^56^, making it a promising candidate for further study. To advance the use of mosquito cells for vaccine production, the cell line must be generated, banked, and maintained under full good manufacturing practices (GMP), a process that entails, among other requirements, comprehensive characterization and safety testing. Even so, our findings indicate that suspension cultivation of mosquito cells offers promising potential for producing biologics made by mosquito-infecting viruses.

## Methods

### Cells and viruses

*Ae. albopictus* C6/36 (ATCC CRL-1660) and U4.4 cells were maintained at 27 °C in adherent cultures using Leibovitz L-15 medium (Gibco) supplemented with 10% heat inactivated fetal bovine serum (FBS; Gibco), 2% tryptose phosphate broth (Gibco), and 1% nonessential amino acids (Gibco). African green monkey kidney Vero E6 (ATCC CRL-1586) cells were cultured at 37 °C and 5% CO_2_ in Dulbecco’s Modified Eagle Medium (DMEM, Gibco), supplemented with 10% FBS, 1% penicillin and streptomycin (Sigma-Aldrich). The Usutu virus (USUV) isolate was obtained from a blackbird (*Turdus merula*) in the Netherlands in 2016 (lineage Africa 3, GenBank accession no. MH891847.1) and propagated on Vero E6 cells. Virus passage 7 (2.86E + 07 TCID_50_/mL) was used for all experiments.

### Suspension cultivation of mosquito cells

Suspension cultivations of C6/36 and U4.4 cell lines were incubated at 27 °C in chemically defined EX-CELL medium (Sigma-Aldrich), supplemented with additional 1% penicillin and streptomycin (Sigma-Aldrich) and 0.1% Pluronic F-68 (Gibco) (hereinafter referred to as EX-CELL medium) at an agitation speed of 110 rpm (Infors HT incubator). Suspension cultures were maintained in 125 mL closed shake flasks (Nalgene) with a 20 mL working volume and a starting inoculation cell concentration of 0.5 × 10^6^ cells/mL.

### C6/36 cultivation in stirred tank bioreactors

The cultivation of C6/36 in a stirred tank reactor was performed in a 500 mL bench-top bioreactor vessel (MiniBio, Applikon), controlled through a control unit (My-control, Applikon) connected to a laptop. Most bioreactor parameters were based on previous work with other insect cells (e.g., Sf9 cells)^57^. The culture was inoculated with a cell concentration of 0.5 × 10^6^ cells/mL (300 mL working volume) in EX-CELL medium and was kept at 27 °C with 300 rpm impeller speed (marine impeller). The SCB IIB sterile tubing welder (TERUMO) was used to weld tubing to and from the bioreactor. The pH was measured with a pH probe (Applisens) and was not controlled. Temperature was measured with a pt100 temperature probe and regulated via an integrated heating pad. DO% was measured using an optical oxygen probe (Applisens) and controlled via macrosparging with air (30% DO). The headspace flow was kept at 3 L/min.

### Cell concentration & viability assay

The cell concentration and viability of a culture were determined by manual counting using C-CHIP disposable hemocytometer chambers (Neubauer approved, NanoEntek) under a light microscope (Leica DMi1). For the viability measurements, dead cells were identified via trypan blue staining. To stain dead cells, a cell suspension sample was diluted 1:1 with 0.4% trypan blue solution (Gibco). The C6/36 and U4.4 suspension cells may grow in moderate-to-large clumps, which can obstruct counting. In that case, samples were vortexed at maximum velocity for at least 10 s to dissociate cell aggregates before standard cell counting.

### Virus infections

C6/36 or U4.4 adherent cell cultures were seeded at 60% confluency (2.5 × 10^6^ cells) and allowed to attach for 2 h in a 6-well plate. After attachment, the cells were infected at a multiplicity of infection (MOI) of 0.1 TCID_50_/cell in L-15 medium. After three hours, the inoculum was removed, the cells were washed with PBS, and fresh medium (EX-CELL or L-15) was added. Time samples were taken at the indicated time points and stored at − 80 °C. The experiment was repeated independently three times.

Infections in shake flasks were performed by inoculating C6/36 cells at a total cell concentration of 0.5 × 10^6^ cells/mL in EX-CELL medium in 125 mL shake flasks (Nalgene, 20 mL working volume) followed by the addition of virus at an MOI of 0.1 TCID_50_/cell. Samples were taken at the indicated time points and stored at -80 °C until virus quantification. Bioreactors were infected in two steps, first a shake flask containing the entire inoculant culture for the bioreactor was infected at a MOI of 0.1 TCID_50_/cell. This flask was then left to incubate for three hours at 27 °C and shaking at 110 rpm. After three hours, the infected inoculant culture was transferred from the shake flask into the bioreactor vessel.

### Virus quantification

Tissue culture infectious dose 50% (TCID_50_) was determined by end-point dilution assays (EPDA). Vero E6 cells from confluent tissue cultures were detached with Trypsin-EDTA (Gibco) and resuspended in a total volume corresponding to 1 mL/1.67 cm^2^. The cell suspension was added to a 10-fold virus serial dilution in a 1:1 ratio. Each dilution was then plated 6 times onto 60-well microtiter plates (Nunc), and virus was scored based on cytopathic effect 5 days post-infection. Titers were calculated using the Reed & Muench method^58^.

### Calculations

Doubling times of suspension cultures were assessed by calculating the specific growth rate in the exponential phase of the growth, as previously reported^59^.

Virus yield was defined as the total virus per cell (TCID_50_/cell) at the time point when peak virus titers were measured.

To estimate the impact of shear forces, the Kolmogorov length was estimated to be around 123 μm through Eq. 1. Where the kinematic viscosity ($\:v$) is equal to 1.0 × 10^− 6^ m^2^/s and the energy dissipation ($\:\epsilon\:)$ is equal to 4.3 × 10^− 3^ m^2^/s^3^.

1 $$\:\eta\:={\left(\frac{{v}^{3}}{\epsilon\:}\right)}^{0.25}$$

## Supplementary Information

Below is the link to the electronic supplementary material.

Supplementary Material 1

Supplementary Material 2

## Data Availability

All data generated or analysed during this study are included in this published article (and its Supplementary Information files).
