# Supplementary figures and images for: Suspension cultivation of mosquito cell lines for the production of the mosquito-borne flavivirus Usutu virus in a stirred-tank bioreactor
Source: Sci Rep. 2026 Jan 9;16:3742. doi: 10.1038/s41598-025-33792-z (PMC12852204; doi:10.1038/s41598-025-33792-z)

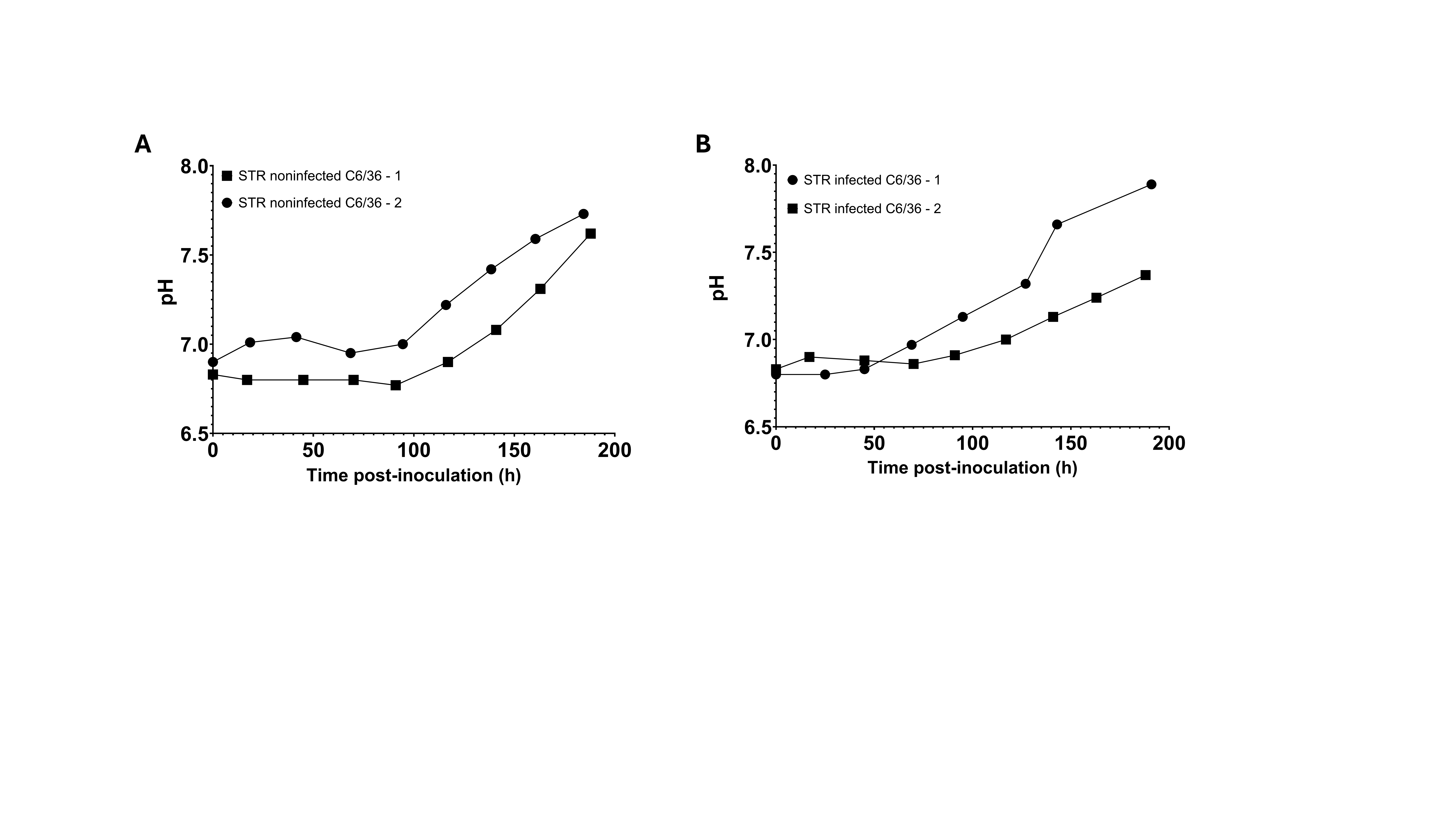

Supplement: Supplementary file 1 — Supplementary Material 1 [file 41598_2025_33792_MOESM1_ESM.tif]
