# Supplementary material for: Suspension cultivation of mosquito cell lines for the production of the mosquito-borne flavivirus Usutu virus in a stirred-tank bioreactor
Source: Sci Rep. 2026 Jan 9;16:3742. doi: 10.1038/s41598-025-33792-z (PMC12852204; doi:10.1038/s41598-025-33792-z)
Supplement: Supplementary file 2 — Supplementary Material 2 [file 41598_2025_33792_MOESM2_ESM.docx]

Supplementary figure 1: EX-CELL medium pH during stirred-tank bioreactor cultivation. (A) pH from both reactor experiments cultivating C6/36 cells. (B) pH during both reactor experiments where C6/36 cells were infected.
